# A web-portal for interactive data exploration, visualization, and hypothesis testing

**DOI:** 10.3389/fninf.2014.00025

**Published:** 2014-03-26

**Authors:** Hauke Bartsch, Wesley K. Thompson, Terry L. Jernigan, Anders M. Dale

**Affiliations:** ^1^Multi-Modal Imaging Laboratory, Department of Radiology, University of California, San DiegoSan Diego, CA, USA; ^2^Departments of Cognitive Science, Psychiatry, and Radiology, Center for Human Development at University of California, San DiegoSan Diego, CA, USA

**Keywords:** data exploration, data sharing, genetics, data dictionary, imaging, hypothesis testing

## Abstract

Clinical research studies generate data that need to be shared and statistically analyzed by their participating institutions. The distributed nature of research and the different domains involved present major challenges to data sharing, exploration, and visualization. The Data Portal infrastructure was developed to support ongoing research in the areas of neurocognition, imaging, and genetics. Researchers benefit from the integration of data sources across domains, the explicit representation of knowledge from domain experts, and user interfaces providing convenient access to project specific data resources and algorithms. The system provides an interactive approach to statistical analysis, data mining, and hypothesis testing over the lifetime of a study and fulfills a mandate of public sharing by integrating data sharing into a system built for active data exploration. The web-based platform removes barriers for research and supports the ongoing exploration of data.

## 1. Introduction

Data exploration is an interactive approach involving extraction of relevant characteristics from complex datasets with the aim of formulating hypotheses that lead to collection of new data and experiments (Tukey, 1980). In order to shorten the time required for producing and confirming novel results the interactive component of data exploration can be implemented as a frequent switching between phases of data exploration for the purpose of generating hypotheses and hypothesis testing. However, without proper statistical tools that implement appropriate tests and control for multiple comparisons, data exploration can easily degrade into data fishing, with poor reproducibility of hypothesis test results in independent samples.

Data exploration can also be useful for data curation, quality control, guidance, and early intervention if applied during the data acquisition phase of a project. Thus, effective data exploration tools can improve data quality by identifying problems of study design or execution in a timely fashion. Furthermore, data exploration tools can facilitate analyses by abstracting them from technical considerations such as data location, how information is encoded and what file formats are used. Diverse data sources such as demographic, neurocognitive, imaging, and genetic information can be analyzed in a unified manner by implementing guidelines for the selection of appropriate statistical models. Providing a system that actively supports data exploration combined with hypothesis testing across data modalities is a valuable adjunct to facilities focused primarily on data sharing like the Neuroimaging Informatics Tools and Resources Clearinghouse (NITRC) (Buccigrossi et al., 2008) and the database of Genotypes and Phenotypes (dbGaP) (Mailman et al., 2007).

### 1.1.Data sources

*Medical imaging* studies collect anatomical and functional volumetric images in search of biomarkers to detect disease or to characterize normal development. Because the pictorial representation of structures in imaging does not easily lend itself to statistical analysis (unstructured data), the imaging data are processed, usually automatically, resulting in structured data with an organization into quantitative measurements for features in regions of interest (Dale et al., 1999; Desikan et al., 2006; Hagler et al., 2009). If image data are acquired by multiple sites each device might introduce systematic variation in the data that can hinder the detection of effects or introduce spurious correlations. Documenting auxiliary measures such as the identity of the imaging scanner (i.e., device serial number) and the version of the software used to perform image reconstruction provides essential additional information that can lead to increased power and accuracy in statistical analyses.

*Demographic information*, *neuromedical history*, and *self-report* measures are all captured by questionnaires and digitized in tabular form which results in a mixture of categorical variables and continuous variables like gender, age, or household income. Information about patient and family history and socio-economic factors provide important context for the interpretation of data from other sources and are often correlated with clinical outcomes (Monzalvo et al., 2012).

*Neurological function* and *behavior* are measured by tests of cognition, emotion, motor function, and sensory function. Standardized tests to obtain these measurements are available (Wechsler, 2004; Weintraub et al., 2013) and can be used to obtain either raw or age-normalized scores.

*High density gene chips* measure variation in single nucleotide polymorphisms (SNPs) in a large number of locations across the whole genome. Typically, on the order of 0.5–2.5 million locations are genotyped for each participant (1000 Genomes, 2012; Fjell et al., 2012). Each location is coded as one of several (two or more) alleles for each study participant. Differences in the frequencies of alleles can be linked to behavioral or structural phenotypes. The distribution of alleles can also be compared to known reference populations, providing information about the genetic ancestry mixture for each participant in the study.

Structured data from these different modalities need to be combined for appropriate statistical analyses that can also control for measured covariates. For example, genetic ancestry may covary with imaging measurements (Biffi et al., 2010), or socio-economic data may covary with cognitive measurements (Hurst et al., 2013). When this information is integrated into the statistical analysis, ancestry admixture effects can be disassociated from effects driven by socio-economic factors.

### 1.2. Applications

Many open-source projects and commercial applications provide support for data acquisition and study control (Wang et al., 2008; Harris et al., 2009; OpenClinica LLC., and collaborators, 2013), storage and sharing of imaging data (Marcus et al., 2007; DCM4CHEE, 2013), viewing collections of images (Rosset et al., 2004; Weasis, 2013), organizing collections of genetic information (Purcell et al., 2007), statistical analysis of cognitive, self-report and psychophysical measurements (JMP, 1989–2007; R Core Team, 2013). One notable difference between these applications and the application presented here (Data Portal) is that the Data Portal promotes integrated data exploration and statistical analyses across behavioral, imaging, and genetics domains.

**Figure 1:**
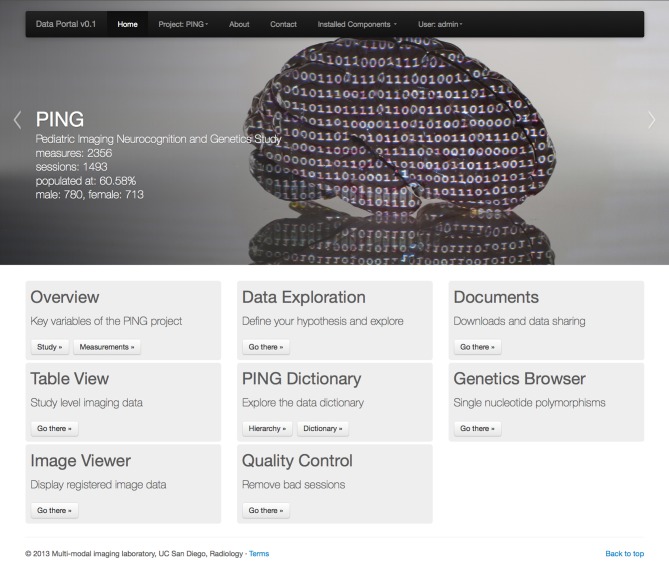
**Entry page to the PING data portal reflecting the architecture of the data portal as a collection of workflow driven components**. A navigation menu structure and project data summary is displayed in the top half of the page followed by a list of eight application groups. See section 2 for a description of each component.

In this paper we describe the features of the data portal for exploratory data analysis and hypothesis driven statistical analysis in the context of the Pediatric Imaging, Neurocognition and Genetics (PING) project (Fjell et al., 2012, http://pingstudy.ucsd.edu, see Figure 1). The PING study contains information from over 1500 subjects between the ages of 3 and 20 years and was created to provide a publicly shared database able to link genetic information and behavioral measures with developing patterns of brain structural connectivity and morphology.

The Data Portal allows for the simultaneous exploration of roughly 2300 distinct morphological, demographic, and behavioral measures as well as 500,000 genetic measures obtained on each study participant. Investigators can define and execute statistical models online for data exploration and hypothesis testing. This makes it possible to discover and explore patterns in multiple data domains while controlling for covariates using a rigorous statistical framework. For a given statistical model the portal also supports the exploration of multi-modal image data for any individual subject. The displayed data include structural magnetic resonance images (MRI), diffusion tensor images (DTI) such as fractional anisotropy (FA), apparent diffusion coefficients (ADC), and directionally encoded color (DEC) images, atlas based fiber tracks and surface reconstructions for vertex (surface point) based measures for cortical thickness, regional surface area expansion and regional volume expansion. The combination of study-level analysis with the capabilities of personalized, participant specific exploration of key developmental features support data exploration efforts especially during the data acquisition phase of a project.

## 2. Materials and methods

### 2.1. Nomenclature

The data portal distinguishes between *projects* as collections of data and *applications* as project neutral entities for data analysis and visualization. This separation supports several projects hosted side-by-side on the same system. Applications use access to project data to implement specific workflows. As an example, the table application can be used to review the registration of diffusion weighted images and structural scans for a large number of subjects. Images are displayed in a table with selected demographic entries for each session. Two example images are displayed in separate columns for each of the structural scans (horizontal section of T1) and the registered diffusion weighted scans (horizontal section of FA). This arrangement of subject information visually highlights any misalignment of images as disagreement of structural information displayed in the two image modalities. A link guides the user to the image viewing application that provides a multi-planar reconstruction of available image volumes.

As a secondary workflow the table application helps to identify image data for a known subject identification number. The user can filter the table columns for subject and visit identification number to identify a particular study session.

Applications implement a restricted set of functionalities but provide interfaces that allow them to exchange information with each other. For example, the table application is able to filter data and provides links to the image viewing application. The image viewing application accepts this information and is able to visualize three dimensional reconstructions of multi-modal images.

For this work we refer to collections of subject data as *sessions*. For example, all image data and all the neurocognitive measures obtained during a single visit are collected into a single session that is identified by the subject's identification number and a visit number or date. Typically, the session information is stored in a single row in a data table. *Measures* identify the quantitative or qualitative data obtained for each session and map to columns in this table. Any measure that is not available for a particular session is left empty.

### 2.2. Technology

The data portal is implemented using a rich client-server, web-based architecture. The web-server delivers data in JavaScript Object Notation (JSON) format together with application code delivered as JavaScript. The clients receive the data, execute the application logic and render the result. Server-side data compression and client-side caching of static data were found to be effective in limiting the resources required on the server (virtual machine with 2GB of main memory and 2 CPU's). The minimum hardware requirements on the client are 1 GHz or faster processor with at least 2 GB RAM and graphics hardware supporting WebGL/OpenGL rendering. The web-interface rendering is implemented using responsive web design and generates appropriate interfaces for workstation computers, laptops, tablet computers, and smart phones. The application has been successfully tested and is functional on all of these device types.

As a general rule all applications transfer project data from the server to the client machine. The client's browser is responsible for filtering and rendering of the data. All modern browsers have advanced built-in capabilities for data caching, which reduces the dependency of the application on network delay because successive requests can be served from the client's cache. The availability of many JavaScript based libraries for data conversion, analysis, and visualization make it straight forward to adapt novel visualization techniques. An example of a JavaScript library that supports many data visualization tasks is D3 (Bostock et al., 2011). Compute intensive applications such as image analysis cannot be efficiently implemented in JavaScript yet (but see first attempts to improve processing speed by ASM, 2013; Pixastic, 2013). Specialized applications for statistical analysis are also not yet available as a component for web-based architectures. For both of these use cases we integrate server-side processing instead. We will mention in each of the following sections if a server-side implementation was selected.

#### 2.2.1. Server

Server side document storage of structured data is done by text files in either JSON format or in comma-separated-values format (csv) which has been selected as a format of lowest common denominator available at the different data acquisition and processing sites. Whereas more traditional relational databases require an interface to import new data into the data model, our simplified approach stores the original data delivered by each site. As such, updates of the data are synonymous with replacing files and data integrity and versioning are implemented by version control software. Additional information such as user logins, project descriptions and project documentation is stored using JSON notation. This notation is compact enough to be efficient for transport, is supported for automatic parsing on both server and client side and can be viewed and edited as text.

Due to the distributed nature of the PING project, with 10 separate data acquisition sites, it was beneficial to keep a separation of imaging-derived measures (termed *imaging spreadsheet*) and measures related to demographic, genetic and cognitive information (termed *super spreadsheet*). This reduced the dependencies between the research groups handling onsite data acquisition and the group responsible for image data processing as they operated on different schedules. Additionally to the imaging spreadsheet and the super spreadsheet each user of the portal can provide a third, private spreadsheet with supplemental data to integrate derived measures or measures not part of the official dataset (such as site-specific additional measures). Merging of spreadsheets is implemented in the R statistical language (R Core Team, 2013) (freely available software), resulting in an efficient binary, user-specific representation of the study data on the server.

Investigators depend on a stable version of the study data for publication purposes. In order to support reference data sets as well as frequent data updates, the Data Portal provides versioning for uploaded data sets. Users can select the currently active version they wish to work with. As selection of the active version is specific to a browser session users can use this feature to document data differences.

#### 2.2.2. Client

Client applications are built using HTML5 (2013) technology supporting modern application interfaces that run inside standard web-browsers. The client has been successfully tested on Internet Explorer 9.0 (and later), Chrome (version 31 and later), Firefox (version 26 and later), Safari (version 7 and later), and Opera (version 18 and later). The user interface is built using the bootstrap front-end framework (Twitter, 2013) with additional jQuery user interface elements (jQuery, 2013). It provides a consistent look and feel across the different Data Portal applications and supports multiple device types and screen form factors.

### 2.3. Statistical analysis

The ability to collate data from multiple sources allows exploration of inter-relationships in the data in a rigorous manner. In order to support online statistical analyses in the Data Portal, we implement an application that combines a web-based interface with server-side statistical processing using R (R Core Team, 2013).

#### 2.3.1. Region of interest based analysis

**Figure 2:**
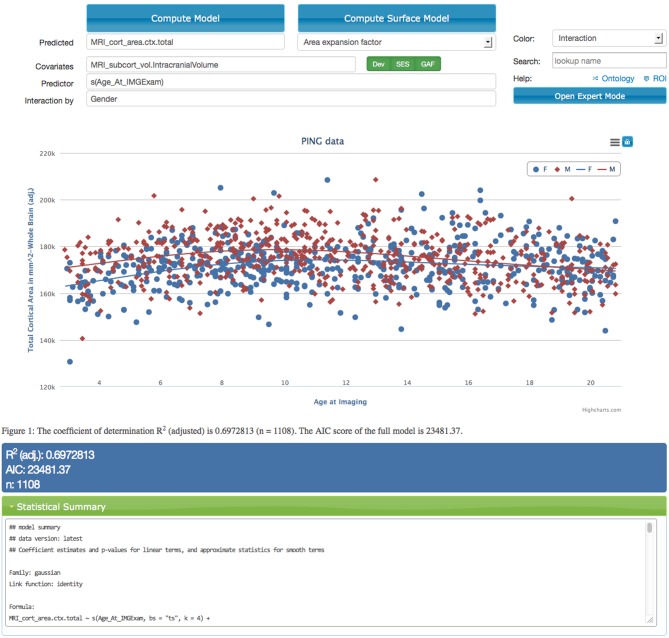
**Screen capture of the data exploration application displaying a statistical analysis of the effects of age on the total cortical area for male (red dots and curve) and female (blue dots and curve) children in the PING study**. The model corrects for the effects of intra-cranial volume, scanning device, socio-economic factors, and genetic ancestry. Interface components that relate to model specification are shown above the scatter plot. The model is executed on the server using R after selecting the “Compute Model” option. Resulting model curves and residualized data points are plotted together with summary statistics in the middle and lower parts of the web-page. The scatter plot supports an interactive legend, changes in magnification, and data points that link back to imaging data.

The application provides input fields organized into a model description mask (see top part of screen capture in Figure 2) that allows the user to specify variables of interest (see section 2.5). The application does not require prior knowledge about the syntax used by the R programming language and provides immediate feedback if terms are entered that are not present in the data dictionary. Descriptions for all terms are displayed as tool tips to the user. The model variables include a dependent variable, an independent variable and an arbitrary list of user defined covariates. Additionally the input mask also supports the definition of a separate variable that should interact with the independent variable. In models that are used to describe interactions it is important to include both the main effect of the interaction variable and the interaction term itself which is automatically the case if the interaction field is used.

Additionally, we identified sources of variation known to influence a variety of measures. These *system covariates* include the device serial number of the imaging device, the household income and level of education as socio-economic factors, and genetic ancestry factors derived from gene expression patterns. Users can disable the system covariates, but they are enabled by default (options displayed in green in Figure 2). Providing these factors is one way in which domain expert knowledge is implemented in the application. Genetic ancestry factors are encoded as probabilities and are therefore dependent on each other. The system thus automatically removes one of the ancestry groups from the analysis to provide the statistical analysis with the correct degrees of freedom. Utilizing meaningful presets and automatic model extensions in this fashion help to make the statistical analysis application accessible to a wider audience.

Regression analyses are performed on the server using a generalized additive model (GAM) framework with automatic smoothness constrains (Wood, 2013). GAMs include usual linear regression as a special case and are applicable to cross-sectional (single time point) analyses. R reads the project data in binary form, executes the imported model and generates summary measures and model comparison statistics as temporary files. Summary statistics together with model curves and data point coordinates are saved as JSON and transmitted to the client, which is responsible for presenting the data to the user.

Suitable matrix formulations for the computationally intensive parts of the R statistical analysis have been implemented to improve performance of the application. As a further optimization the analysis is restricted to session data for which all model variables are non-missing; sessions with missing values are removed as a first step in the analysis. It is possible in principle to do an initial multiple imputation step for missing data, but this is not currently implemented. The resulting independent and dependent variables are rendered by the client as an interactive scatter plot. Axis labels are inserted using the short description obtained from the data dictionary application, and each data point can be queried using the mouse to display its value and basic demographic information such as gender and age. A link presented to the user for each data point provides a direct connection to the image viewer application that loads relevant session images. Together with the scatter plot, model curves (GAM fits) are displayed in order to provide feedback to the user about the relationship between the dependent and independent variables, including interaction terms. For example, if age is used as an independent variable and gender is used as an interaction term, separate mean curves for males and females are displayed. If the effect of the predictor variable is modeled as a smoothly varying function, the model curves might indicate gender specific changes in the predicted variable. The freedom to specify arbitrary variables of interest makes this statistical framework suitable for a wide number of research questions related to age trajectories of brain development. As an example in section 3 we show how to use the PING data portal to analyze the influence of socio-economic factors on imaging measures while correcting for age, gender and genetic factors.

Together with a visual representation using scatter plots and model fits, the application also displays the statistical summary information computed by R (lower part of Figure 2). This includes the version number of the data, the generated model specification and the *p*-values for each of the factors. Key model characteristics such as Akaike (1974) and Schwarz's Bayesian information criteria (Schwarz, 1978) are displayed as well and can be used to compare models with different covariates with each other. Both of these model selection procedures help guard against over-fitting by inclusion of too many variables with small effects.

In order to document a particular model users can either export scatter plots in image or spreadsheet format or users can download the data and the R script used for processing. This information can be used to document findings, and users with appropriate knowledge of the statistical models can also alter the script. During development this feature helped in detecting errors created, for example, by inconsistent encoding of measures in the spreadsheets.

#### 2.3.2. Surface based analysis

**Figure 3:**
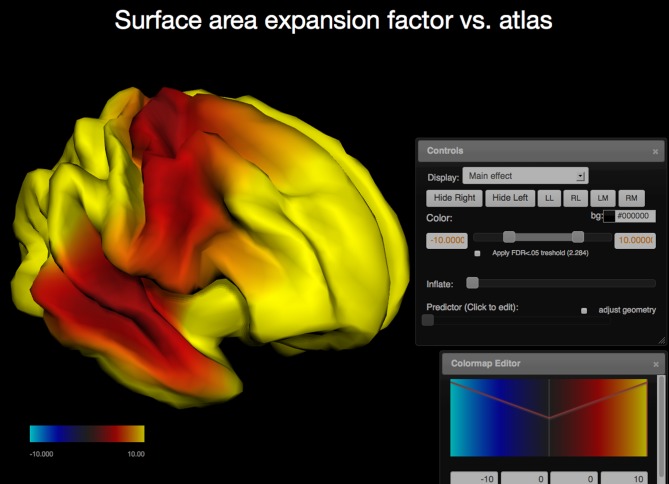
**Screen capture of the surfer viewer application**. Color is used to map the −log_10_(*p*) values of the main effect of age onto each vertex (WebGL cortical surface rendered on the left, same statistical model as in Figure 2). The two user interface components displayed are the Colormap Editor (bottom right) which controls a step-wise linear colormap and the “Controls” interface (middle right) that provides a selection of main and interaction effects as well as an option to display the predicted values for each vertex over the range of the predictor (age). Further options include surface re-orientation, background color selection, control of the false discovery rate to correct for effects of multiple comparisons, and an option to adjust the geometry as a predicted variable.

In addition to region of interest based measures derived from imaging data, the PING study also produced surface based measures for cortical thickness, regional area expansion, and regional volume expansion for each study participant. In this mode the vertex measures are used as dependent variables and the R model is run for each vertex. The resulting surface maps represent regional effect sizes for the (1) user-defined independent variable, (2) the main effect of the interaction variable, if any, (3) its interaction with the independent variable, and (4) estimates for the dependent variables per vertex over the range of the independent variable. Surface maps are written out as JSON and requested by the client. The client renders the surfaces interactively and maps the *p*-values as color (Cabello, 2013; WebGL, 2013); animated maps are used to show the values of the dependent variable over the range of the predictor. The brain geometry is rendered as two independent hemispheres and the user interface provides keyboard shortcuts to allow for the inspection of the inter-hemispheric space (see Figure 3).

By default surface maps are rendered using a static surface geometry derived from an atlas brain. The application also provides an option to calculate and display the geometry as a predicted variable. In this mode the surface geometry is deformed to show the shape trajectories of predicted variables such as age corrected for influences of the selected covariates.

Performing the same statistical analysis for each vertex requires multiple-testing corrections for tests of significance. The application provides for a correction for multiple comparisons using the false-discovery rate (FDR) (Benjamini and Hochberg, 1995). The client uses this information to adjust its color mapping for *p*-value maps using neutral gray tones for regions that are not deemed significant. The user has control over the color mapping and can adjust the colors in the application using step-wise linear transfer functions. Users may also select a point on the surface using the mouse. The name of the corresponding closest region of interest is displayed in that case together with a highlight that shows the outline of the region.

### 2.4. Viewing images

**Figure 4:**
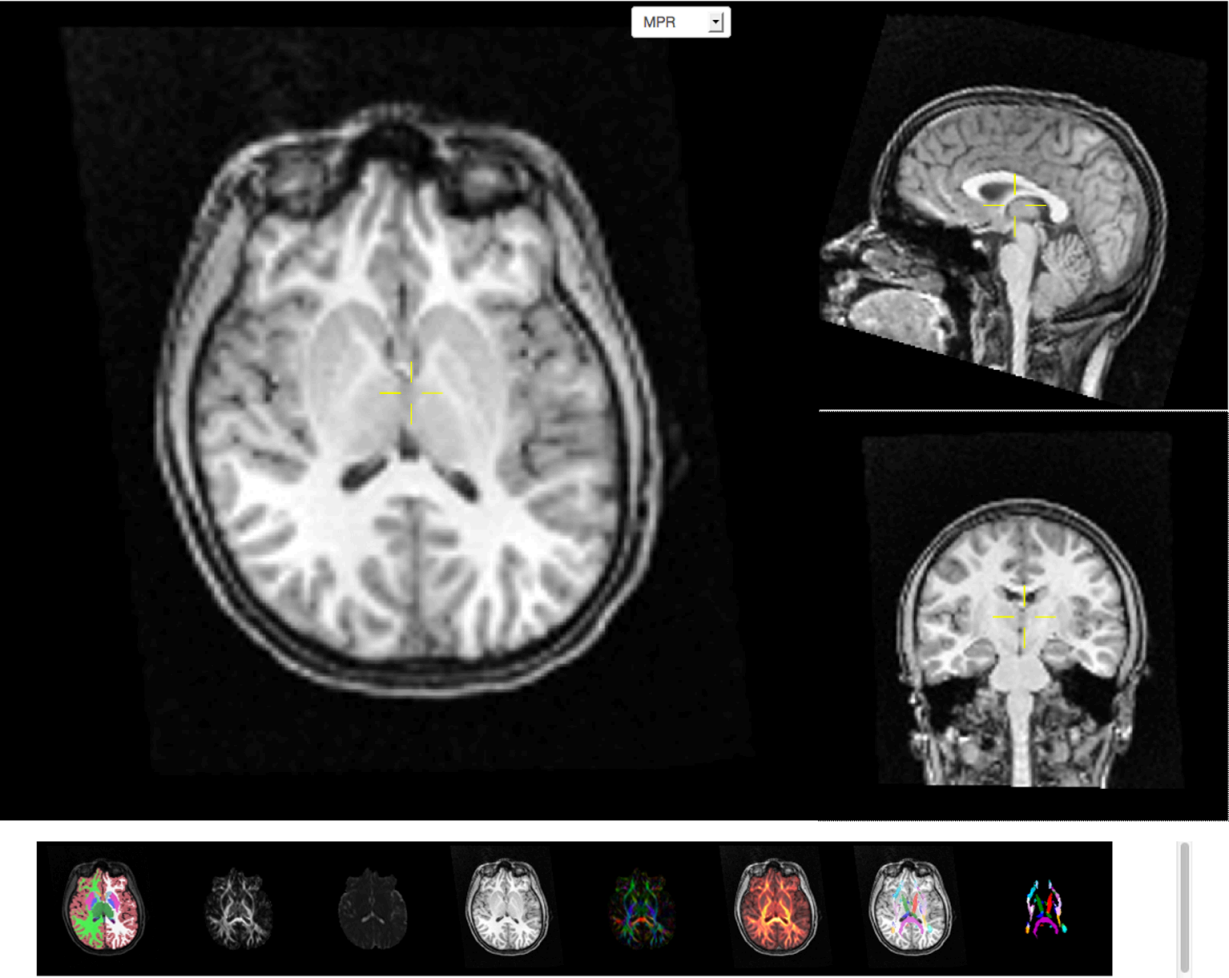
**Screen capture of the image viewer application**. A multi-planar reconstruction displays axial (top left), sagittal (top right), and coronal (middle right) images linked by a common cross-hair (pale yellow). Below, a row of axial thumbnail images depict available image modalities such as (left to right) fused sub-cortical segmentation with T1-weighted anatomical image, fractional anisotropy (FA), mean diffusivity, T1-weighted anatomical image, color coded directional image stack, fused FA and T1 image stack, fused fiber atlas tract with T1 and fiber atlas tract image stack. All image modalities are registered with each other and selection of a thumbnail image will display the corresponding volumetric information in the multi-planar viewer component above the row of thumbnails. All images support slice browsing using the mouse wheel, brightness, and contrast calibration, and image zoom.

Image data are often acquired in search of biomarkers for diseases. Such biomarkers are derived from anatomical and functional images and are screened by statistical methods for effectiveness in diagnosing disease. In order to get reliable, observer independent measures, automated atlas based image processing pipelines are used (Dale et al., 1999; Hagler et al., 2009). Quality of the generated data depends on appropriate scanning sequences and adherence to scanning protocols. In order to detect protocol violations and other technical anomalies automated and manual quality control of images and derived segmentations are required. This control step is used to identify cases that have to be rejected due to artifacts created for example by subject motion, incorrect scanner settings, or signal dropout. The image viewer application (see Figure 4) supports such a quality control workflow by providing a direct link between raw image data and volumes derived after automatic registration and processing. As an example T1 weighted intensity images and color coded cortical and sub-cortical labels are fused together to allow for a visual inspection of cortical segmentation relative to anatomical scans of T1 image intensity. Scans derived from diffusion weighted imaging are also available as overlays onto anatomical images which supports the inspection of multi-modality registration procedures.

The image viewing application presents a multi-planar reconstruction of volumetric data that is displayed as linked coronal, axial and sagittally oriented images for each modality. A cross-hair tool is used to identify a 3D location in each image stack and the corresponding two orthogonal images closest to this location are loaded from the server and displayed. Scrolling also requests new image tiles from the server. The image storage on the server contains the images for all three orientations registered across several image modalities for each session. The client application displays thumbnail images for each image modality available and switching between modalities allows the user to inspect a specific image location across image modalities. The viewer supports image zoom and pan operations and provides basic adjustments for contrast and brightness. On-demand loading of image tiles and browser caching of already downloaded images allow for interactive performance over a pre-computed image cache containing several millions of images.

### 2.5. Data dictionary

We define a data dictionary as an organization that structures technical terms and their textual descriptions. It is used in this work as the basis for a machine interpretable and processable documentation of the PING related technical terms. The primary use of this resource is to allow researchers not familiar with the PING project to identify measures of interest. The scope of the PING data dictionary is restricted to terms that describe imaging, demographic, self-report, neurocognitive, and genetic measures.

The initial form of the data dictionary is created from the column headers of the data spreadsheets gathered by different units in the research study. Each entry is used as a category (term) with two attributes, a short description which is suitable as an axis label and a textual explanation describing the data encoding in detail. This textual information is made available as a web-service, and data portal application such as the statistical analysis tool utilize this resource.

**Figure 5:**
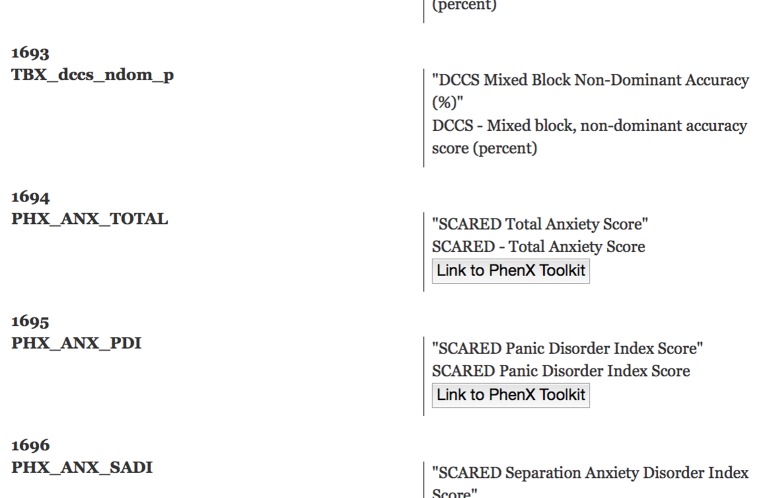
**Screen capture of a section of the data dictionary displaying NIH toolbox measures**. A sequential number is displayed together with the dictionary term on the left side of the page. On the right side, the corresponding axis label (top) and the available long description (bottom) is listed. Links to external resources such as the PhenX toolkit are embedded into the page. This HTML5 encoded document also contains the RDFa structure information to facilitate knowledge extraction.

The data dictionary application provides two visual representations of the data dictionary. All terms are displayed as a list in the *data dictionary view* using HTML5 with embedded RDFa (W3C, 2012, see Figure 5). This structured representation allows for data integration and reasoning using external tools. Furthermore, where appropriate, the list displays links to external resources such as the PhenX toolkit (Hamilton et al., 2011). The *structured graph view* facilitates the data exploration of the data dictionary terms. In the PING study more than 2300 measures are available for each study session. Browsing through this collection of terms is supported by imposing a structure that links related terms. The linkage is not exhaustive but merely done in an effort to balance the displayed hierarchy in terms of the number of hierarchy levels and the number of leaf nodes in each category.

We define a term as either a string of characters that is taken from the initial data dictionary or a grouping term that is accompanied by a pattern that maps the grouping term to a subset of all terms. Patterns are implemented as regular expressions utilizing linguistic relationships between terms similar to the work of Ogren (2004). The PING data dictionary lends itself to this analysis as it contains many terms that are derived hierarchically using a small sub-set of root strings. For example, 700 imaging related terms contain the initial string “MRI_” followed by a categorization of the measurement type as either cortial area, thickness, volume, or contrast, followed by an indication for left or right hemisphere and a string characterizing the name of the region of interest. Further examples include self-report measures using the PhenX toolkit (Hamilton et al., 2011) that start with the string “PHX_”, genetic ancestry measurements starting with “GAF_”, and cognitive measurements obtained using the NIH cognitive toolbox (Weintraub et al., 2013) that start with the letters “TBX_”.

Regular expressions are a flexible and efficient way to test large collections of strings. But term clustering using string matching methods is known to be only efficient for small sets of patterns (Tanaka, 1995). Currently about 100 patterns are used to describe the balanced hierarchical structure of the PING project data dictionary. A main requirement for global pattern matching to work is that the string representation of terms used throughout the project is unique. In certain cases we found that terms shared the same name, e.g., “Age” was used to indicate the age at imaging examination and also in a separate group to indicate the age at the neurocognitive examination. In an effort to resolve these cases new terms were introduced to make the entries unique (e.g., “Age_At_IMGExam”, “Age_At_NPExam”). This approach to normalization is clearly inefficient and further efforts are needed to include relational types between categories as well as attributes for synonyms and abbreviations. In most cases, if new entries are added to the data dictionary and if those new entries follow already established naming conventions, no change in the pattern set is required to integrate the measurements into the structure. To validate correctness, a coverage check is performed to ensure that (1) the new term is matched by a pattern and (2) appears in the correct place in the hierarchy. If the new term is not correctly matched but conforms to the naming conventions the list of patterns is changed. Changes include the extension of existing patterns to cover the new term using alternations and the introduction of new categories as they are required.

**Figure 6:**
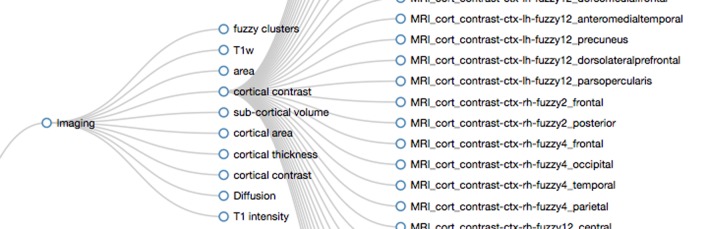
**Screen capture displaying parts of the hierarchical structure of the PING data dictionary**. The branches for “Imaging” and “cortical contrast” have been opened by the viewer. The regular expression used to create the displayed hierarchy level for “Imaging” is “/(H_area|H_thickness|H_contrast|H_volume|H_intensity|Diffusion|H_Fuzzy)/”. The entry “cortical contrast” (H_contrast) is implemented by the pattern “/(^MRI_cort_contrast)/”. In PING this maps to all MRI related cortical contrast measures in the data dictionary (subset displayed on the right).

In order to visually represent the derived structure, we use an interactive graph layout (Bostock et al., 2011) which adjusts if elements are added or removed (see Figure 6). Only the first two levels of the hierarchy are displayed initially. The user can select a term, the corresponding pattern is executed, and the hierarchy level below the selected term is populated with the matching entries. The layout engine adds the structure using animated unfolding and adjust the spacing between terms. Exploration of the data dictionary structure is therefore interactive and efficient as it only depends on the parts of the hierarchy that the user explores.

### 2.6. Genetic information

Genotyping using microarrays generates vast amounts of data for each study participant. The size of a typical data vector for each study participant is in the order of 500,000 or more elements. Each of these vectors consists of allele combinations of many SNPs located across the genome. SNP location is specified in terms of number of base pairs from the start of the chromosome. SNP location may overlap with functional regions of the genome that encode genes, pseudo-genes, non-coding RNA, or mRNA sequences. A typical approach to browsing genome-wide data is to search for a particular gene based on findings that relate this gene to a function of interest. SNP values that are captured by the study and which overlap with, or are close to, the gene of interest are selected and used in statistical analyses as independent variables.

**Figure 7:**
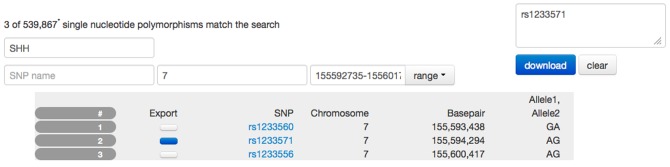
**Screen capture of the SNP browser application used to explore and extract genetic information available for the PING study**. A search mask is used to specify a gene (SSH, sonic hedgehog). Utilizing a database with 80,000 entries, the SNP browser obtains the available chromosome number (7) and the basepair location (155,592,735–155,601,766) for this gene. The table is filled with SNP entries that fall in the range of the basepair location. In this example, three SNP entries are available. The user has selected SNP number 2 indicated by the dark blue checkbox and the corresponding SNP name has been copied to the list of SNP names for download. Selecting the download option would provide the user with a spreadsheet of the alleles for this SNP for all PING subjects.

The SNP browser provides a user interface that links together gene names and their locations on the chromosome, as well as SNP locations and allele combinations for each study participant (see Figure 7). We found that network speed and modern browser technology easily keep up with the transmission of data generated by larger studies. Data can be stored on the client computer in memory but browser-based applications are limited in terms of their ability to simultaneously display graphical representations often involving thousands of objects. We solve this problem by using client-based logic that prevents content from being rendered that is not currently visible in the browser window. User interactions like scrolling are used as a signal to add content. As an example, the SNP browser application appears to list initially all 500,000 SNP's in a single table as no search term is specified at the start of the application. As the user scrolls down the page, data are dynamically added to the bottom using unobtrusive pagination. This approach limits the number of items rendered in the browser and adjusts naturally with differences in screen size and resolution of client machines.

The user can search for a specific gene using its name or a suitable regular expression that is matched against all gene names. The client application filters the global set of names and populates the chromosome and the base pair range fields in the interface. This search is performed on the client computer using cached data and does not require resources on the server or even a connection to the server. The resulting table displays the subset of SNP locations that fall into the base pair range indicated by the gene. Each of the SNPs displayed is linked to the NCBI (2013) database for further information.

The SNP browser provides access to the study specific SNP data so users can select candidate SNPs for further analysis. The SNP names are collected as a editable list in the interface and, upon request, the list of SNP alleles for each subject id is generated and presented to the user as a csv spreadsheet for download. Due to the size of the SNP database (approximately 2 gigabytes of binary data) a server-side implementation using the *PLINK* software (Purcell et al., 2007) is used to create each spreadsheet. PLINK provides an efficient binary storage for large samples of SNP data which reduces storage requirements and, more importantly, provides a fast read and access to SNP information. Updates of SNP data are supplied as PLINK files, which are copied directly to the server. No further processing is required to integrate the information into the Data Portal. Processing time on the server is similar to the time required to download the resulting spreadsheet.

The SNP browser exports data that are suitable for upload into the statistical analysis application of the Data Portal. Together with genetic ancestry information already available in the statistical analysis application, this setup provides a flexible solution allowing genetic information to be linked to other data domains.

### 2.7. Quality control

Several applications are suitable to detect outliers in the data. For example, the image viewing application shows region of interest (brain labels) merged with anatomical information. Errors in the segmentation are easily identifiable on these images. Outliers on the population level are apparent in the scatter plots of the data exploration application. Each data point in scatter plots links to the corresponding imaging data. Exploring these data can increase confidence that observed variation is due to true variation between study participants and is not caused by differences in image quality or noise levels.

A dedicated application is used to capture the current status of quality control. Sessions with known problems are indicated and textual annotations are used to explain choices of inclusion or exclusion of data from analysis. Access to the quality control application is limited to trained personnel using a role-based access control system. If a data point is marked as “bad,” it is excluded from further analysis by forcing a re-generation of the data cache available to each user. Results of the process are immediately accessible to every user of the portal.

### 2.8. Security and privacy considerations

Removal of patient identifying data is performed during data acquisition as stipulated by the responsible institutional review boards. Only limited information is provided to researchers requesting access. However, the combination of many sources of information such as genetic, demographic, and imaging data poses unique challenges for data privacy.

For example, genomic data have the potential to link people across studies (Homer et al., 2008). These data could therefore be used to re-identify study participants or their relatives. In order to prevent such activities and to ensure the privacy of study participants, access to the Data Portal is secured. Each user of the Data Portal is required to agree to a data use policy that forbids the use of study data for the purpose of de-identification. As a further precaution the SNP viewer implements limits on the number of SNP values that a user can download for a given project. The download of all SNP values is not supported by the data portal but may be provided using dedicated data sharing sites for genetic information like dbGAP (Mailman et al., 2007).

## 3. Results

The large number of variables available in the PING study provide a rich resource for data exploration of patterns of brain development. As an example we show how to explore a measure of cortical surface area over age. Area of the heavily folded human cortex correlates under some circumstances with the number of neurons available for processing and has been implicated as a variable of interest for describing the developing human brain.

*Identifying variables of interest:* The first step is to identify data dictionary entries that refer to the variables of interest. Using the data dictionary application a measure of the total cortical thickness called “MRI_cort_area.ctx.total” can be found in the sub-tree *imaging*. Additionally, the entry is also listed in the section labeled *summary measures*. If parts of the variable name are known the data exploration application input fields can be used as search fields that display the matching content in drop-down lists. Entering the search strings “area” or “total” would list the measure together with a textual description as a tool tip.

Initially we start with the simplest model by disabling the system defined covariates that represent imaging device identity, socio-economic factors and genetic ancestry factors. After specifying the independent and dependent variables in the data exploration application as total cortical area and age at image examination the model is executed and produces a linear functional relationship between cortical surface area and age. This initial model shows a large spread of the scatter points which indicates a poor explanatory power of the model which is confirmed by a low value of variance explained (0.076%) as listed in the statistical summary section. Only a small part of the relationship between brain surface area and age can be explained by our initial model.

*Model comparison:* Replacing the linear function of age “Age_At_IMGExam” with a smoothly varying function “s(Age_At_IMGExam)” we can improve the fit. The new model captures an initial increase in total cortical area followed by decreasing cortical area over age (variance explained 7.5%).

Adding back the sequence of system covariates for imaging device, socio-economic factors (household income, highest level of parental education) and genetic ancestry the models can capture successively more of the variance (13, 17, 20%). It is well known that adding variables to a model will tend to increase its explanatory power which at some point will lead to poor generalization as accidental features of the data are captured. Also, as new measures are added a subset of subjects will need to be removed from the analysis if measurements are not available for them. In order to be able to detect over-fitting the data exploration application displays the adjusted coefficient of determination *R*^2^ which incorporates a correction for the number of variables included into the model. Increasing values over successive runs of the model confirm that our model variables help to explained the observed variance without introducing over-fitting. This is also confirmed by the displayed Akaike Information Criterion (AIC, Akaike, 1974).

Whereas the system covariates have been identified as sensible choices for model testing it is up to the investigator to identify further variables. A potential source of variation not captured by our current model is gender differences. Also, cortical area will likely scale with head size so our measure for total surface area includes effects that can be attributed to varying head sizes. Identifying measures for gender and intra-cranial volume and including them into the model increases variance explained to 70%.

*Significance analysis:* The significance of each model variable is listed in the statistical summary section of the data exploration application. The socio-economic factors for example show that effects of household income are significant (*p* < 0.01) whereas the level of the highest parental education is not.

*Analysis of interaction:* Adding gender as a covariate explained a large part of the variance observed. This could indicate that there is substantial difference in the developmental patterns of male and female children. In order to investigate these differences the data portal can calculate interaction effects with age and display separate model curves for each gender. Moving gender from the covariate text field to the field labeled *interaction* both the main effect of gender and the interaction effect of gender and age are added to the model. We observe a highly significant interaction effect of age by gender (*p* < 0.001). The total cortical area over age curves generated by executing this model suggest that the developmental trajectories differ for boys and girls. Cortical area appears to peak slightly earlier and decline somewhat more rapidly in boys than in girls.

*Adding data sources:* Using the rich literature of genes implicated in development and disease we can use the portal to try to replicate findings using the PING data. The STON2 gene has been implicated as being correlated with regional surface area in a model of schizophrenia Xiang et al. (2013). The SNP Browser application of the Data Portal provides access to selected SNPs that are located in genes of interest. A search for “STON2” reveals three SNPs that are located on chromosome 14 and intersect with STON2. Upon request the application generates a csv formatted spreadsheet with three columns of SNP alleles for each PING subject for download. The spreadsheet format is such that it can be uploaded into the data exploration application as a user defined spreadsheet. Adding the SNP names as new covariates we can create a model that tests for significance of SNP allele combinations related to STON2 on total cortical area. Running the extended model reveals no significant effect of STON2 SNP alleles on total cortical area in the PING study. Other external sources of subject specific information can be integrated into the data exploration portal in a similar manner.

*Surface based analysis:* Cortical area measures are available in the PING study as atlas-based regions of interest measures (Desikan et al., 2006). Additionally, area measures are available for each point on the cortical surface. These measures are calculated as factor values of local area expansion required to map points in the individual brain onto points in an atlas brain. Regions of the brain that need to expand if mapped to the atlas can therefore be distinguished from regions that would need to contract.

The surface based analysis uses the same model description and statistical tools as the region based analysis. Our current model description can therefore also be used to calculate developmental effects of regional surface area. The surface viewer application displays the calculated *p*-values as surface maps of −log_10_ scaled *p*-values (see Figure 3). The scaling is used to map high significance (low *p*-values) to large false-color encoded values indicating regions which are significant at a level of *p* < 0.05. Running the model simultaneously for each point on the surface may cause some regions to reach significance due to chance alone (multiple testing). To counteract this artificial inflation of significance the surface viewer application performs re-scaling of the *p*-value surface maps using false-discovery rate (FDR) calculations. Similar features are available for surface-based estimates of cortical thickness and volume.

Inspection of the calculated point-wise surface area expansion factor due to age shows a general increase for all points relative to the given atlas. This is explained by the choice of the atlas which represents an adult brain. A measure independent of the choice of the atlas is available in the surface viewer application as the instantaneous rate of change. This measure highlights cortical areas that show increased (red to yellow) or decreased (blue) developmental change at a particular age (dependent variable of the model). Using this option the surface viewer animates the complex pattern of developmental change over age for both hemispheres and provides presets for the visual inspection of both hemispheres and the inter-hemispheric spaces.

*Export:* The graphic system used to display the model and scatter plot adjusts to the display size of the device used to view the page. Model curves and point sets can be switched on and off independently using controls embedded in the legend of the figure. Each scatter plot point can be investigated and reveals session information such as the anonymized subject identification number, the gender and links to the image viewer application.

The scatter plot and model functions can be exported as vector graphics (pdf format) or, as quantitative data (csv format) for further statistical analysis. The data exploration application also provides a download package that contains the source code of the statistical analysis and the study data in R format. This package can be used for both documentation and replication of the implemented statistical methods.

**Figure 8:**
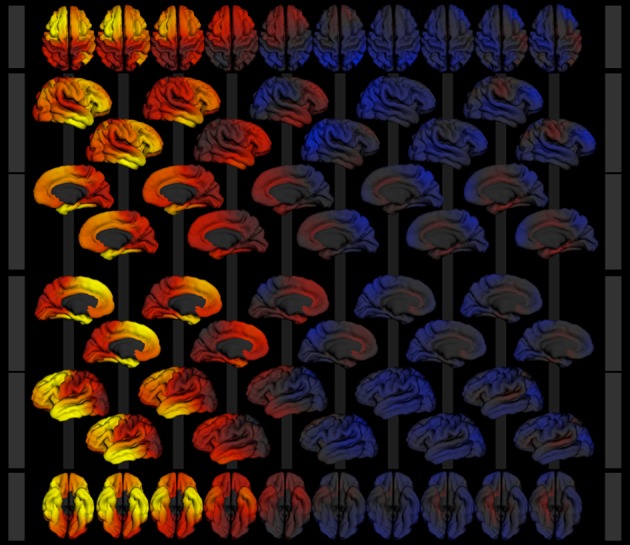
**Image collage of surface models exported from the surface viewer application for the model described in section 3**. Cortical area expansion factor is mapped as color (red—expansion, blue—contraction) over age (3–21 years, left to right) given the model described in section 3. Rows show superior (1), right lateral (2 and 3), medial view of the right hemisphere (4 and 5), medial view of the left hemisphere (6 and 7), left lateral (8 and 9), and inferior (10) views of the 3d surface model.

Each map displayed by the Surface Viewer can be exported as a high quality graphic (png image format) with transparency used to encode background pixel. The graphics can easily be assembled into publication ready representations of key developmental figures (see Figure 8 for image collage).

Efficient implementation of statistical methods on the server reduces the response time for a full statistical analysis to approximately 5–10 s. The combination of automatic model generation for the R programming language, server side execution, export of result data and the integration of visualization and data exploration provides a low barrier of entry for people with limited technical expertise. The features implemented in the data portal therefore extend the usability of study data to a larger audience.

## 4. Discussion

Traditional approaches for data management have used database management system to store all information related to a project. In this setup data import and export algorithms become the tools to map domain specific data to structures suitable for database storage and retrieval. The specific choice for the database layout, such as the number of tables and the values, keys and indexes that are stored in a relational database is expected to be stable over time. This requires projects to make decisions early on, often using insufficient information. Using relational databases therefore can be costly if changes in the database layout are required. Often application logic for import and export of data is used instead to adjust to changing requirements.

The data portal architecture improves on this approach by favoring file formats that are linked to data processing and visualization. These data structures augment the database management infrastructure as a primary source for algorithmic processing. For example, binary representations of large datasets such as genomic data combine compact representation and guarantee fast access while minimizing system resources. Our approach of keeping established data formats such as PLINK's binary format and R's RData format for storage on the server has also simplified the incremental update of our system to new versions of the data because only a small number of files have to be replaced using a very simple procedure.

The PLINK and R software applications are publicly available and provide efficient read access for compact data caches minimizing server requirements on memory and speed. Furthermore, data are stored in a way that is best suited to the specific application that implements the data analysis. Instead of data warehousing with complex implementations of data access, domain specific languages provide an integration for suitable analysis algorithms. In this framework the domain languages are responsible for translation of processing results into formats suitable for transfer and decoding on the client side (JSON, csv).

One of the more challenging aspects of the data portal development has been the efficient transfer of large assemblies of image data. The application preserves bandwidth by downloading only images that are displayed and images that are immediately adjacent. One way to increase viewing performance further is to combine images into mosaics. This would result in a lower number of file transfers with larger files which is more efficient in the setting of web-based applications.

The statistical processing is optimized for cross-sectional studies. Longitudinal analysis requires the use of an extended statistical modeling framework, such as generalized additive mixed models. Currently the data portal is able to detect longitudinal data and data points in the scatter plot that belong to the same participant are marked. A warning is presented to the user that informs him or her about the restrictions imposed by the cross-sectional analysis stream.

The data portal combines the elements of study participant specific exploration of key developmental features across behavioral, imaging and genetics domains with capabilities to formulate and test study level hypotheses and estimate population parameters.

### Conflict of interest statement

The authors declare that the research was conducted in the absence of any commercial or financial relationships that could be construed as a potential conflict of interest.
